# Supplementary material for: Early Prediction of Sepsis in the ICU Using Machine Learning: A Systematic Review
Source: Front Med (Lausanne). 2021 May 28;8:607952. doi: 10.3389/fmed.2021.607952 (PMC8193357; doi:10.3389/fmed.2021.607952)
Supplement: Supplementary file 1 [file Table_1.pdf]

SUPPLEMENTAL DATA

Supplementary Table 1. Search strategy for each database on July 20th, 2020

Bibliographic database	Search String	Remarks
EMBASE	`(``sepsis prediction'' OR ``sepsis detection'') AND (``machine learning'' OR ``artificial intelligence'')`	
Google Scholar	`(``sepsis prediction'' OR ``sepsis detection'') AND (``machine learning'' OR ``artificial intelligence'')`	Software 'Publish or Perish' used to perform the search
Pubmed/Medline	`(``sepsis prediction'' OR ``sepsis detection'') AND (``machine learning'' OR ``artificial intelligence'')`	
Scopus	`TITLE-ABS-KEY ((``sepsis AND prediction'' OR ``sepsis AND detection'') AND (``machine AND learning'' OR ``artificial AND intelligence''))`	
Web of Science	`TOPIC: ((``sepsis prediction'' OR ``sepsis detection'') AND (``machine learning'' OR ``artificial intelligence''))` Timespan: All years. Indexes: SCI-EXPANDED, SSCI, A&HCI, CPCI-S, CPCI-SSH, BKCI-S, BKCI-SSH, ESCI, CCR-EXPANDED, IC.	

Supplementary Table 2. List of variables extracted

Category	Variable	Remark
Publication Details	Author<br>Year of Publication<br>DOI	
Demographics	Sex<br>Age<br>Ethnicity<br>Number of patients with sepsis<br>Number of controls<br>Prevalence of sepsis	count<br>years<br>count<br>count<br>count<br>%
Study details	Dataset<br>Prediction task<br>Sepsis defintion<br>Case-control alignment<br>Inclusion Criteria	 <br><br>Alignment of sepsis onset in controls
Software and Data	Data of used cohort available<br>Code for analysis<br>Code for sepsis label<br>Software<br>Library versions<br>Code licence available	 <br><br><br><br>yes/no
Machine learning details	Model used<br>Model explanation<br>Evaluation metrics<br>External validation<br>Handling of missing data<br>Data types<br>Number of variables<br>Handling of circularity	

Supplementary Table 3. An explanation of quality assessment criteria

Categories	Items	Description	Reported
Unmet need	Limits in current non-machine-learning approach	Low diagnostic accuracy, low human-level prediction accuracy or prolonged diagnostic procedure	Yes/No
Reproducibility	Prevalence of Sepsis	The proportion of patients that sustained a sepsis out of the entire study cohort	Yes/No
	Data availability	Is the data used in the study publicly available?	Yes/No
	Feature engineering methods	How features were generated before model training	Yes/No
	Code for data wrangling and analysis	Code describing the details of the cleaning, preprocessing, and analysis of the data.	Yes/No
	Code of label	Code describing sepsis label generation	Yes/No
	Platforms/packages	Both platforms and packages should be reported	Yes/No
	Hyperparameters	All hyperparameters that are needed for study replication	Yes/No
Robustness	Sample size > 50	Sepsis case sample size > 50 is required for the interpretation, power, and validity of machine learning methods.	Yes/No
	Valid methods for over-fitting	Valid methods for unbiased performance assessment (or methods that prevent overfitting)	Yes/No
	Stability of results	Calculated variation in the validation statistics	Yes/No
Generalisability	External data validation	Validation in settings different from the research framework	Yes/No
Clinical significance	Predictors explanation	Explanation (biological or quantification) of the importance of each predictor	Yes/No
	Suggested clinical use	Clinical usability and requirements (e.g. what is still necessary before making deployment possible)	Yes/No

Supplementary Table 4. Demographic information reported by all studies (if available)

	Study	Age	Sex (% female)	Ethnicity (% non-Caucasian)
1	Abromavicius 2020	—	—	—
2	Barton 2019	55 (median, USCF), 65 (median, MIMIC-III)	54.62 (USCF), 43.87 (MIMIC-III)	—
3	Bloch 2019	55.4 (target group mean), 52.5 (control group mean)	35 (target group), 40 (control group)	—
4	Calvert 2016	—	—	—
5	Desautels 2016	65 (median)	43.85	—
6	Futoma 2017	—	—	—
7	Kaji 2019	—	—	—
8	Kam 2017	—	—	—
9	Lauritsen 2020	—	—	—
10	Lukaszewski 2008	60.16 (mean)	42.42	—
11	Mao 2018	65 (median)	43.97	—
12	McCoy 2017	—	51.43	—
13	Moor 2019	67.2 (target group mean), 64.2 (control group mean	41.4 (target group), 45.4 (control group)	27.9 (target group), 28 (control group)
14	Nemati 2018	—	52.4	—
15	Reyna 2020	—	—	—
16	Schamoni 2019	63 (median)	43.39	—
17	Scherpf 2019	—	—	—
18	Shashikumar 2017a	59 (median)	47	—
19	Shashikumar 2017b	63 (median)	48.8	—
20	Sheetrit 2019	—	38.4 (target group), 42.2 (control group)	—
21	van Wyk 2019a	—	—	—
22	van Wyk 2019b	—	—	—
